# Comprehensive review of penile cancer using MR imaging

**DOI:** 10.1186/s13244-025-02089-0

**Published:** 2025-12-19

**Authors:** Océane Charret, Claire Faget, Thibaut Murez, Juliette Coutureau, Ingrid Millet

**Affiliations:** 1 Department of Medical Imaging, Lapeyronie University Hospital, Montpellier, France; 2 Department of Urology, Lapeyronie University Hospital, Montpellier, France; 3 Desbrest Institute of Epidemiology and Public Health (IDESP), Univ Montpellier, INSERM, Montpellier, France

**Keywords:** Penile cancer, MRI, Tumor staging, Imaging protocol, Surgical planning

## Abstract

**Abstract:**

Magnetic resonance imaging (MRI) is considered the gold standard for staging penile squamous cell carcinoma and assessing its extent. However, due to the rarity of this pathology, few medical centers have regular experience with penis carcinoma imaging. The purpose of this article is to provide a comprehensive update on the role of MRI in penile cancer by reviewing the MRI anatomy of a normal penis, outlining the recommended MRI techniques for penis assessment, and discussing the benefits and drawbacks of artificial erection. We will also highlight how MRI can serve the purpose of tumor staging and its therapeutic consequences.

**Critical relevance statement:**

To provide a comprehensive and practical review of penile cancer based on imaging, including epidemiology, prognosis, treatment, penile MRI protocol, anatomy, and key points for accurate analysis.

**Key Points:**

- Penile carcinoma affects the glans and/or the foreskin in 98% of cases.
- MRI is the most accurate imaging modality for staging penile carcinoma and assessing its extent.
- T2-weighted using thin section is the best sequence to identify the tumor.
- Accurate treatment depends on the depth of local invasion and lymph node involvement.

**Graphical Abstract:**

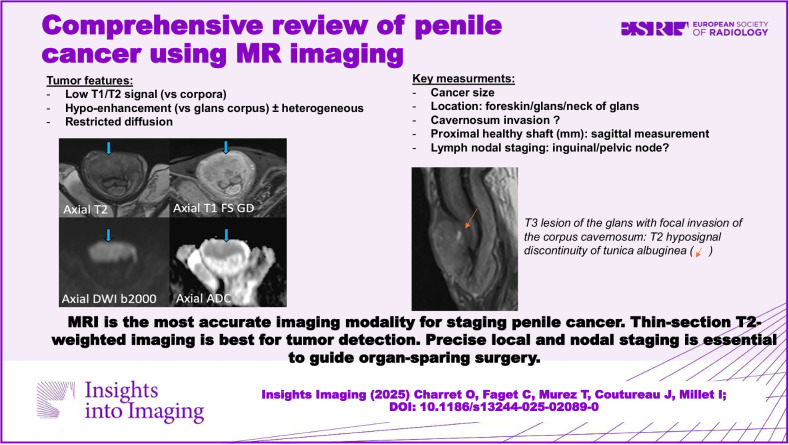

## Introduction

Penile cancer assessment based solely on clinical findings often results in inaccurate staging and suboptimal treatment. Indeed, compared to physical examination, MRI has proven to be superior in assessing the depth of the invasion of the cavernosal bodies [1], a key factor in planning treatment and penile preservation. Imaging, especially MRI due to its superior soft tissue contrast [2], plays a vital role in tumor-node-metastasis (TNM) staging and treatment planning, including tumor resection and lymph node management.

Until recently, imaging was performed after a diagnostic biopsy, if the clinician suspected deep extension. However, the recent European Association of Urology (EAU) guidelines on penile cancer [3] recommended a dedicated penile MRI before biopsy to rule out the invasion of the cavernous bodies, especially when penile preservation is planned. Magnetic resonance imaging (MRI) can also provide useful information regarding resectability in case of large (T4) tumors with invasion into adjacent structures [3].

Penile cancer, although rare—with an overall incidence of < 1.00/100,000 males in Europe and the USA [4]—primarily affects men over 40 [5–7], with a rising incidence linked to Human papillomavirus (HPV) infection [3]. The incidence of penile cancer in men varies significantly across regions, with the highest rates observed in less developed nations. In some Asian, African, and South American countries, the incidence of penile cancer constitutes up to 10% of malignancies in men [8]. Diagnosis is often delayed due to a gap between symptom onset and consultation, primarily caused by patient embarrassment.

Approximately one-third to half of penile cancer cases can be attributed to HPV-associated carcinogenesis, especially types 16 and 18 [6, 9, 10]. Other risk factors include phimosis, lack of circumcision, poor hygiene, chronic inflammatory conditions (such as balanitis), lichen sclerosus, low socio-economic status, multiple sexual partners, treatment with psoralen or ultraviolet A photochemotherapy, and smoking [11].

The majority (95%) of primary penile cancers are squamous cell carcinomas (SCC) [12]. Penile intraepithelial neoplasia (PeIN) is considered the precursor lesion of penile SCC. There are different subtypes of penile SCC classified by the World Health Organization (WHO, 2022) into HPV-associated and HPV-independent subtypes [13]. Histological grading follows the WHO/International Society of Urological Pathology system: Grade 1 includes well-differentiated cells with minimal atypia; Grade 3, predominantly anaplastic cells; and Grade 2, intermediate tumors. The differential diagnosis of penile SCC includes basal-cell carcinoma, malignant melanoma, mesenchymal tumors, sarcoma, extramammary Paget’s disease, urethral carcinoma, lymphomas and metastases, which are extremely rare in comparison to SCC [3, 4, 9, 14] and will be briefly discussed at the end of this article.

The main prognostic factors are the degree of deep invasion by the primary tumor and the status of the draining lymph node [15, 16]. Staging is then critical, as prognosis depends on these factors. Early-stage cancer (I–II) has a 5-year survival rate exceeding 85% with appropriate treatment, while advanced stages with pelvic lymph node metastases have survival rates below 10% [15, 17, 18].

This article provides a comprehensive review of penile cancer, focusing on MRI protocols, key diagnostic points, and treatment implications.

## Staging

The 8th edition of the Union for International Cancer Control (UICC)/American Joint Committee on Cancer (AJCC) (UICC/AJCC) TNM is the classification system used currently for penile cancer, which was last updated in 2017 (Table 1). In this version, invasion of the corpus cavernosum is considered T3 disease, and urethral involvement is no longer deemed relevant to local staging.

**Table 1:** TNM clinical and pathological classification of penile cancer according to the UICC/AJCC 8th edition

T: Primary tumor (same classification for clinical and pathological assessment)	
Tx: Primary tumor cannot be assessed T0: No evidence of primary tumor Tis: Carcinoma in situ Ta: Noninvasive localized SCC T1: Tumor invades subepithelial connective tissue T1a: Tumor invades subepithelial connective tissue without lymphovascular invasion or perineural invasion and is not poorly differentiated T1b: Tumor invades subepithelial connective tissue with lymphovascular invasion or perineural invasion or is poorly differentiated T2: Tumor invades the corpus spongiosum with or without the urethra T3: Tumor invades the corpus cavernosum with or without the urethra T4: Tumor invades other adjacent structures	

N: Regional lymph nodes	
**Clinical**	**Pathological (based on biopsy or surgical excision)**
cNx: Regional lymph nodes cannot be assessed cN0: No palpable or visibly enlarged inguinal lymph nodes cN1: Palpable mobile solitary inguinal lymph node cN2: Palpable mobile multiple or bilateral inguinal lymph nodes cN3: Fixed inguinal nodal mass or pelvic lymphadenopathy, unilateral or bilateral	pNx: Regional lymph nodes cannot be assessed pN0: No Regional lymph node metastasis pN1: Metastasis in one or two inguinal lymph nodes pN2: Metastasis in more than two pN3: Metastasis in pelvic lymph node(s), unilateral or extranodal extension of regional lymph node metastasis

M: Distant metastasis	
M0: No distant metastasis M1: Distant metastasis	

## Clinical assessment

The presenting symptoms include redness or induration of the penis, a chronic nonhealing ulcer, or a polypoid growth [9, 19]. The tumor may be obscured by a phimosis.

The initial workup for penile cancer starts with a physical examination, which consists of the visual inspection and palpation of the penile lesion, and an assessment of regional lymph nodes to determine local staging, following the National Comprehensive Cancer Network standard of care.

Clinically, penile cancer presents as a palpable, visible lesion on the penis, characterized as nodular, ulcerative, or fungating. SCC of the penis affects the glans and/or the foreskin in 98% of cases (the glans (48%), the foreskin (21%), glans penis and foreskin (9%), coronal sulcus (6%), and shaft (2%)) [9]. A lesion affecting solely the cavernous bodies of the penis should initially suggest another diagnosis (e.g., metastasis).

Clinical staging is prone to error and can be subjective, often resulting in understaging of the disease, due to difficulties in reliably assessing the depth of invasion [20]. Moreover, for patients with a high body mass index or previous inguinal surgery, physical examination may not be a reliable method to assess disease extent.

Between 30% and 60% of patients with penile cancer present with palpable inguinal lymph nodes. However, it does not necessarily indicate tumor invasion, since secondary infection of the lesion, which is often ulcerated, is common. In approximately half of these cases, the palpable inguinal lymph nodes reflect metastatic lymphadenopathy, while in the other half, they indicate reactive lymph node enlargement. Moreover, up to 25% of patients with penile cancer have non-palpable lymph nodes that harbor metastases [3, 19, 21].

Primary diagnosis is made by clinical examination and biopsy. MRI is then indicated to assess tumoral infiltration of the corpora cavernosa, spongiosum, and urethra, to help the urologist choose the most adequate surgical approach. Additionally, MRI is used for lymph node staging [22, 23].

## MRI

### Accuracy of MRI (primary tumor)

The recent EAU guidelines [3] have recommended a dedicated penile MRI to exclude tumor invasion of the cavernous bodies, especially if preservation of the penis is planned.

A recent meta-analysis [24], which included 8 studies and 481 patients, showed that MRI staging of penile cancer could be considered to differentiate ≤ T1 vs ≥ T2 disease (sensitivity 86% and specificity 89%), although it did not appear more accurate than clinical staging (*p* = 0.83). According to EAU guidelines, MRI does not outperform physical examination in this case.

For the diagnosis of T3 disease, MRI had a sensitivity and specificity of 80% (95% CI: 70–87%) and 96% (95% CI: 85–99%), respectively, which showed that MRI was particularly useful when organ sparing was planned [24]. These results were comparable to those of other studies [25, 26].

MRI also has a very good correlation with histopathological findings: a recent study reported a strong and significant correlation between MRI and histopathology for the largest tumor diameter and infiltration depth (*p* < 0.001) [27].

In stage T4, MRI can provide useful information about the resectability of cancers that are invading adjacent structures.

### Technique

#### Prostaglandin injection: For or against?

According to the last EAU recommendations, injection of prostaglandin is an option. According to a recent study [24], MRI with and without artificial erection showed similar accuracy in local staging.

The choice is therefore left to each team of radiologists in consultation with the urologists.

Some studies [25, 28–30] have suggested that using MRI of the erect penis after an injection of prostaglandin E1 into the corpora cavernosa could improve local staging by providing a better assessment of the depth of invasion. The erect penis is easier to visualize in multiple planes, and its anatomy is often more precisely delineated [2, 31].

Some teams do not routinely image the erect penis for several reasons [18, 32], summarized in Table 2. Others will use intracavernosal prostaglandin selectively in cases where the initial examination, performed without prostaglandin, was insufficient [29, 33].

**Table 2:** Drawbacks of intracavernosal injection of prostaglandin E1

• Complicates workflow and requires more stringent patient screening
• Time spent checking for contraindications and carrying out the injection
• Time between the injection and obtaining an erection (generally 10–15 min)
• Risk of priapism (1% in the Linet et al study) [36]
• Penile pain after injection (in 11% of patients) [36]

#### How to inject prostaglandin if used

This is generally achieved by direct injection of Prostaglandin E1 into the corpora cavernosa to promote smooth muscle relaxation and distention of the erectile bodies [30]. An artificial erection is preferred over a natural one due to the duration of the MRI examination, with a 20-mg dose of intracavernosal prostaglandin inducing an erection lasting an average of 44 min [27–38]. Sildenafil, combined with subsequent manual stimulation by the patient, is an alternative option. However, it has a longer onset time and provides less reliable results [1, 37].

Table 3 shows the main contraindications to prostaglandin injection [1, 24, 27, 37].

**Table 3:** Contraindications to prostaglandin injection

• Allergy to prostaglandin
• Conditions that predispose to priapism: sickle cell disease, multiple myeloma, leukemia, polycythemia
• Penile prosthesis
• Penile fracture
• Cavernosal thrombosis

Care should also be taken in patients with recent vascular disease, treatment predisposing to bleeding, or Peyronie’s disease.

**Fig. 1:**
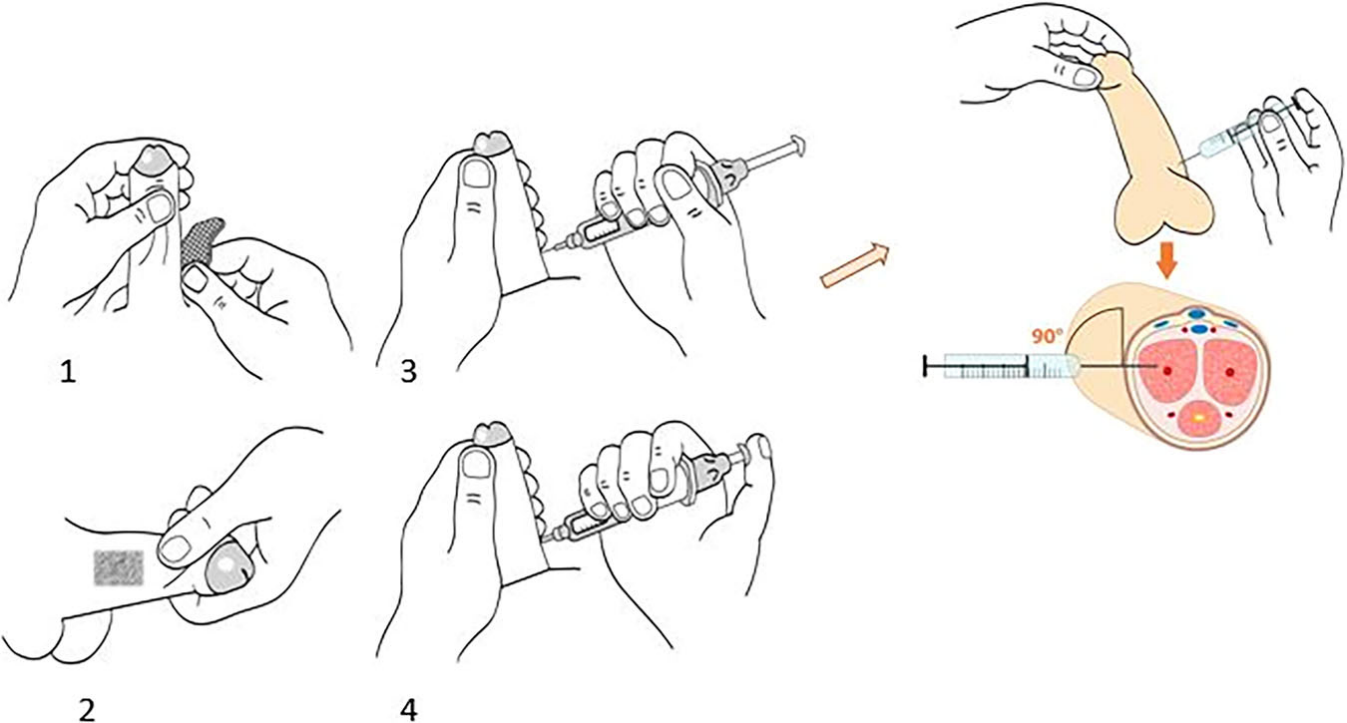
Prostaglandin injection technique. 1 Skin disinfection using alcohol at 90 °C. 2 The penis is stretched to expose the corpus cavernosum. 3 and 4 Prostaglandin is injected with a needle perpendicular to the skin at the 1/3–2/3 junction of the penis

Figure 1 shows the different stages in the injection of prostaglandin.

#### Patient positioning

**Fig. 2:**
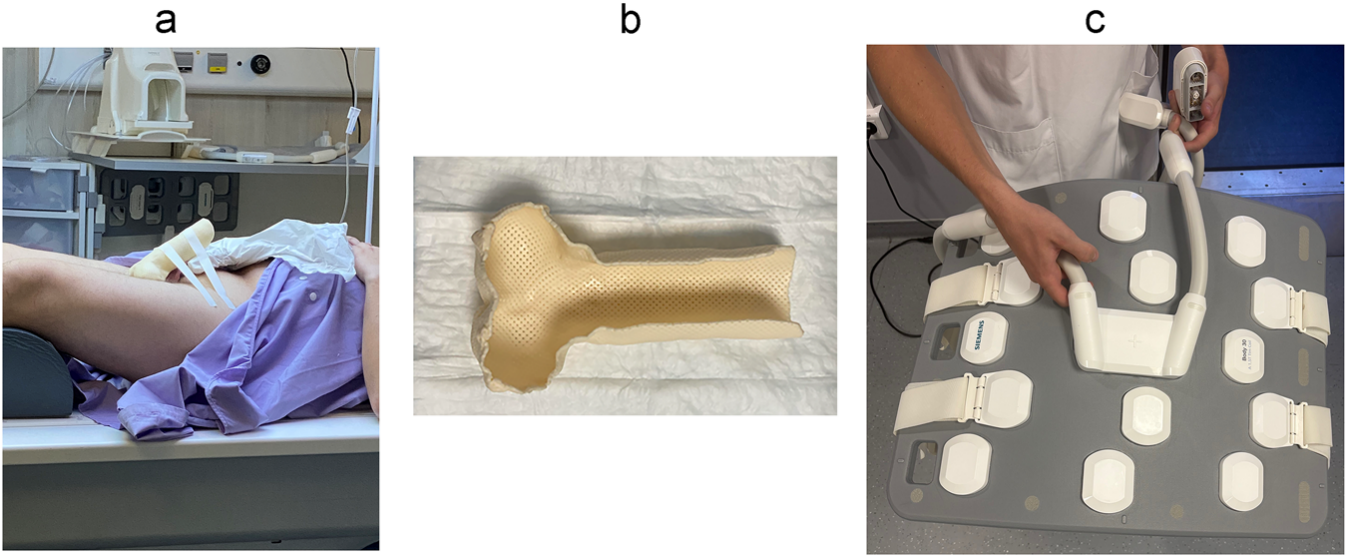
Recommended patient positioning (**a**), the penile thermo-formed shell used in our department (**b**), and surface coil placement (**c**)

The patient is in a supine position with the penis dorsiflexed against the lower anterior abdomen (Fig. 2a), which allows optimal anatomical alignment and coil proximity, despite a potential increase in respiratory artifacts.

In our center, we use a thermoformed shell to improve stability and reduce motion artifacts, achieving very good results (Fig. 2b). A multiple phased-array surface coil is placed on the penis to acquire optimal-quality, high-resolution images (Fig. 2c).

If the dorsiflex position is not possible, the penis should be taped in a midline position to reduce motion during the examination.

A towel is placed between the patient’s legs, beneath the scrotum, to elevate the scrotum and penis [18, 22, 34].

Since 98% of tumors occur in the distal portion of the penis, it is recommended to avoid positioning the penis too closely against the pelvic region, as this may compress or flatten the distal portion.

#### MRI protocol

Table 4 displays the main MRI sequences used in our center, along with those from two other studies for comparison [35, 36].

**Table 4:** MRI protocol for assessment of primary penile carcinoma in our center and in two other studies [35, 36]

	Our center 1.5 T with DL (Siemens)		Switlyk et al [35] 1.5 T with DL (Siemens)	Lubner et al [36] 1.5 T or 3 T
T2W (pelvis)				
Pulse sequence	Blade		−	
Plane	Ax			
Slice thickness (mm)	3.5			
FOV (cm)	26			
Gap (mm)	0.0			
Matrix size				
T2W (penis)				
Pulse sequence	TSE	TSE	−	2D fast SE
Plane	Sag. Cor	Ax		Sag. ax
Slice thickness (mm)	2	3.5		2.4
FOV (cm)	24	24		26
Gap (mm)	10	10		0.2
Matrix size	230 × 256	230 × 256		384 × 256
T2W (tumor)				
Pulse sequence	TSE		2D SE	−
Plane	Ax		Sag, cor, ax	
Slice thickness (mm)	2		2	
FOV (cm)	24		23	
Gap (mm)	10		0.0	
Matrix size	230 × 256		288 × 288	
DWI (penis)				
Pulse sequence	FS Zoomit B50. B1000		Zoomit Reduced FOV EPI B0. B800. B1400	2D B50. B500. B800
Plane	Ax		Sag, cor, ax	Ax. Sag
Slice thickness (mm)	3.5		2.3	4
FOV (cm)	24		15 (limited to the tumor)	32
Gap (mm)	10		0.58	0.2
Matrix size	68 × 152		102 × 102	128 × 128
DWI (pelvis)				
Pulse sequence	–		STIR-EPI B0.B800	–
Plane			Tra	
Slice thickness (mm)			5	
FOV (cm)			25 × 37	
Gap (mm)			0.0	
Matrix size			84 × 124	
T1W before GD (penis)				
Pulse sequence	3D T1 VIBE		−	3D SGRE FS
Plane	Ax			Ax, sag
Slice thickness (mm)	0.8			2.2
FOV (cm)	28.5			26
Gap (mm)	20			0.2
Matrix size	230 × 256			280 × 224
DCE-MRI (tumor)				
Pulse sequence	–		3D spoiled GE Dixon	–
Plane			Ax	
Slice thickness (mm)			2.2	
FOV (cm)			200	
Gap (mm)			0.44	
Matrix size			128 × 128	
T1W after GD (penis)				
Pulse sequence	3D T1 VIBE		–	3D SGRE FS
Plane	Ax			Ax, sag
Slice thickness (mm)	0.8			2.2
FOV (cm)	28.5			26
Gap (mm)	20			0.2
Matrix size	230 × 256			280 × 224
T1W after GD (pelvis)				
Pulse sequence	3D T1 VIBE		3D spoiled GE Dixon	3D SGRE FS
Plane	Cor		Ax	Ax
Slice thickness (mm)	1.5		1.4	3
FOV (cm)	45		30 × 36	38
Gap (mm)	20		0.28	0.2
Matrix size	175 × 288		367 × 416	280 × 224

*DL* deep learning, *FOV* field of view, *DWI* diffusion weighted imaging, *GD* gadolinium, *DCE-MRI* dynamic contrast enhanced MRI, *TSE* turbo spin echo, *VIBE* volume-interpolated breath-hold examination, *SE* spin echo, *SGRE* spoiled gradient echo, *FS* fat sat, *EPI* echo-planar imaging, *STIR* short tau inversion recovery, *GE* gradient echo, *Ax* axial, *Sag* sagittal, *Cor* coronal

T2-weighted sequences are the most important MR imaging sequences for staging tumors due to their high sensitivity. It is crucial to acquire T2-weighted sequences in all three planes, as the lesion and its extension may be best visualized in only one of the planes, depending on the tumor location, especially in the glans area. Non-fat-suppressed TSE T2-weighted sequences should be performed in the axial, coronal, and sagittal planes, using thin Section (2 mm), a small field of view, and relatively high resolution to ensure the best delineation of the tumor.

Sometimes, the tunica albuginea at the glans cannot be easily distinguished from the subepithelial connective tissue, making it difficult to differentiate between T1 and T2 lesions. In that case, if the depth of the extension remains unclear on the standard T2 images, a very thin T2 section can be added in the most representative plane for this clinical situation.

Axial diffusion-weighted images are also obtained using a small field of view. In our center, we use the Zoomit technique, which helps reduce distortion artifacts caused by the magnetic susceptibility. Some centers prefer to restrict the diffusion-weighted imaging field of view to the tumor (cf. Table 4, [35]).

Pre- and post-contrast fat-saturated T1 images, acquired after administering gadolinium at a dose of 0.1 mL/kg, can also be useful.

A wide field of view sequence to assess the pelvic lymph nodes should be included in any MRI protocol [37].

### MRI anatomy of the penis (Fig. 3)

The penile shaft comprises three erectile structures: the paired dorsal corpora cavernosa and the ventral midline corpus spongiosum, which houses the urethra and extends distally to form the glans penis. The glans is the distal continuation of the corpus spongiosum.

**Fig. 3:**
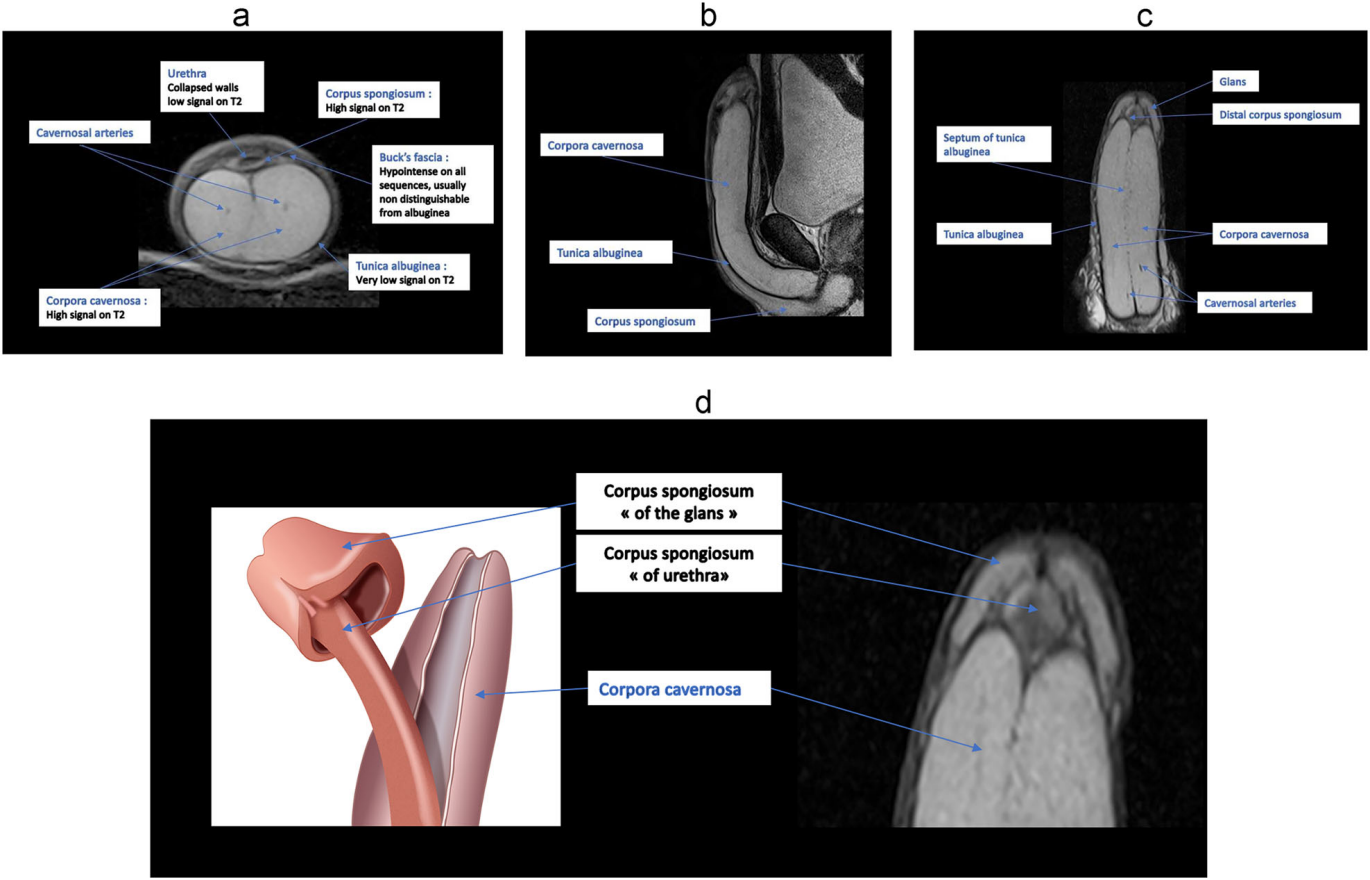
Normal penile anatomy using MRI. T2-weighted images, respectively, on an axial view (**a**), sagittal view (**b**), coronal view (**c**), and coronal view focused on the glans part (**d**)

The penis corpora are encased in three connective tissue layers:

- Tunica albuginea: a fibrous sheath surrounding the corpora cavernosa and corpus spongiosum;
- Buck fascia: a less dense layer closely attached to the tunica albuginea;
- Dartos fascia: the superficial layer, enclosing loose subcutaneous tissue.

**Fig. 4:**
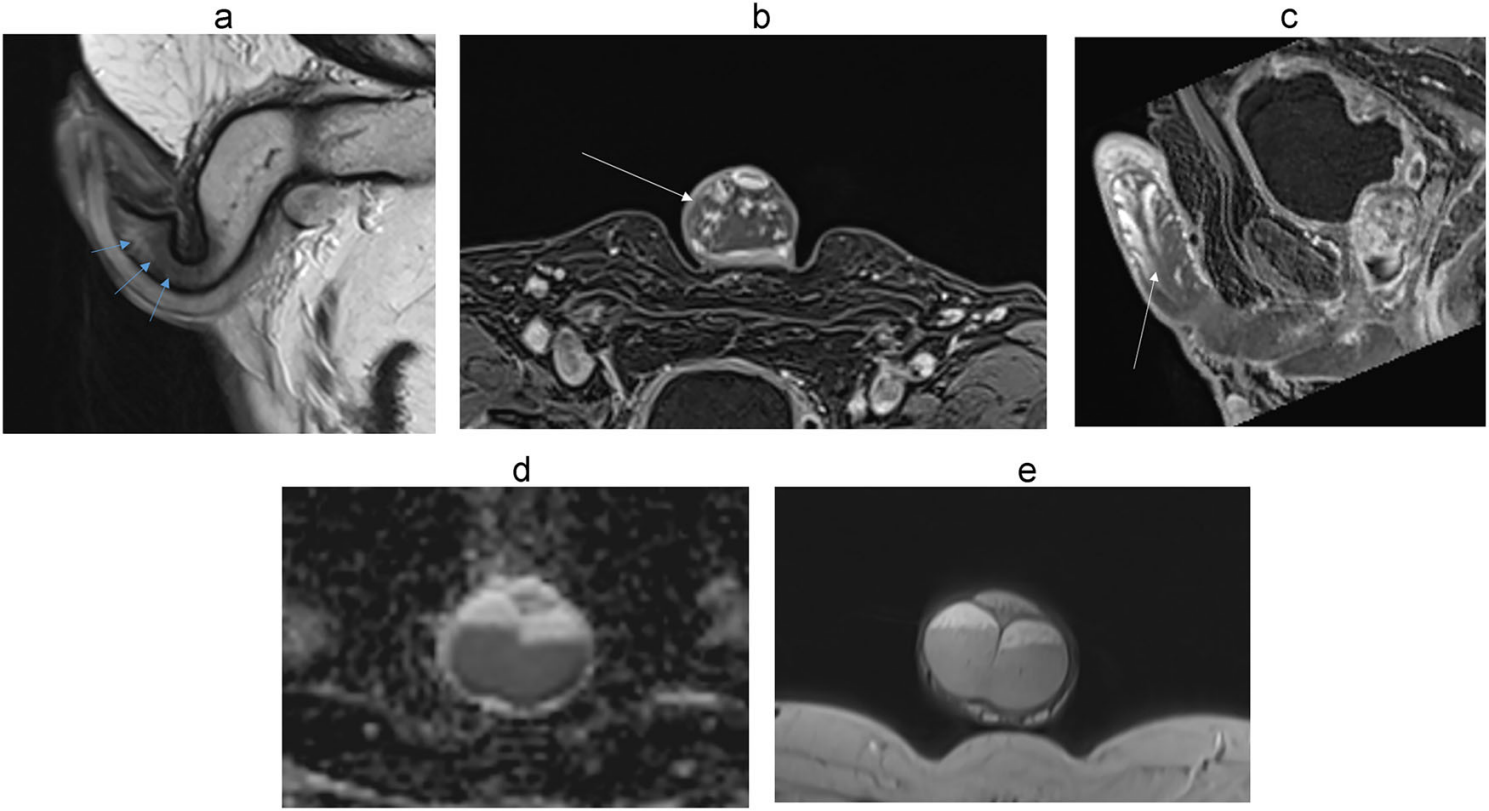
Common imaging pitfalls: T2 hyposignal areas in the flaccid penis (**a**), progressive enhancement of the corpora cavernosa in the axial plane (**b**) and sagittal plane (**c**), blood layering artifact on diffusion (**d**) and T2-weighted sequence (**e**)

On MRI, the corpora cavernosa and the corpus spongiosum show intermediate signal intensity on T1-weighted images and high signal intensity on T2-weighted images. T2 hyposignal areas are common in the flaccid penis and are non-pathological (Fig. 4a); they are often mistaken for fibrosis [28]. Imagining the erect penis can avoid this signal abnormality. The gadolinium-enhanced T1-weighted image shows that the corpus spongiosum enhances almost immediately, while the corpora cavernosa enhance gradually, in a centrifugal manner, due to the central location of the cavernosal artery, leading sometimes to heterogeneous enhancement of the cavernosal bodies (Fig. 4b, c). The layering of blood within the corpora cavernosa is a normal finding when imaging the tumescent penis [32, 38] (Fig. 4d, e), corresponding to a fluid level between the plasma superiorly and the red blood cells inferiorly.

On diffusion-weighted images, the corpora cavernosa and spongiosum are usually hyperintense.

The cavernosal arteries can be seen in the middle of each corpora cavernosa as a T2 hypointense dot [30].

The walls of the urethra, contained within the corpus spongiosum, are T1 and T2-hypointense [18, 33, 38, 39].

Both the tunica albuginea and the Buck fascia appear as a single thick rim with sharp T1 and T2 hyposignal relative to the erectile tissue. MR imaging is not reliable for distinguishing between the tunica albuginea and the adjacent Buck fascia [2, 18, 31, 33, 40].

The subcutaneous connective tissue superficial to Buck’s fascia contains the deep dorsal vein, the Dartos fascia, and the skin epidermis, which appear hyperintense on T2 imaging [2, 41]. Following gadolinium administration, the fascial layers of the penis do not enhance [2, 38]. The deep dorsal vein is usually T2 hyperintense due to slow blood flow.

### Imaging primary epithelial tumors of the penis

#### Tumor staging

The exam begins before the MRI. Radiologists are more likely to detect the tumor on MRI if they know where it is, and they will interpret the findings with greater confidence if they can correlate MRI images with clinical findings. The first step is to identify the tumor’s location visually, through clinical examination, without palpation.

In general, T2-weighted MR imaging sequences are the most useful in defining the local extent of a penile neoplasm.

On MR imaging, penile cancer usually appears as a solitary, ill-defined infiltrative mass that is moderately hypointense on both T1- and T2-weighted images, which makes it possible to distinguish it from the relative hypersignal of the corpora cavernosa. The tumor usually exhibits higher signal intensity than the tunica albuginea on T2-weighted images [31].

**Fig. 5:**
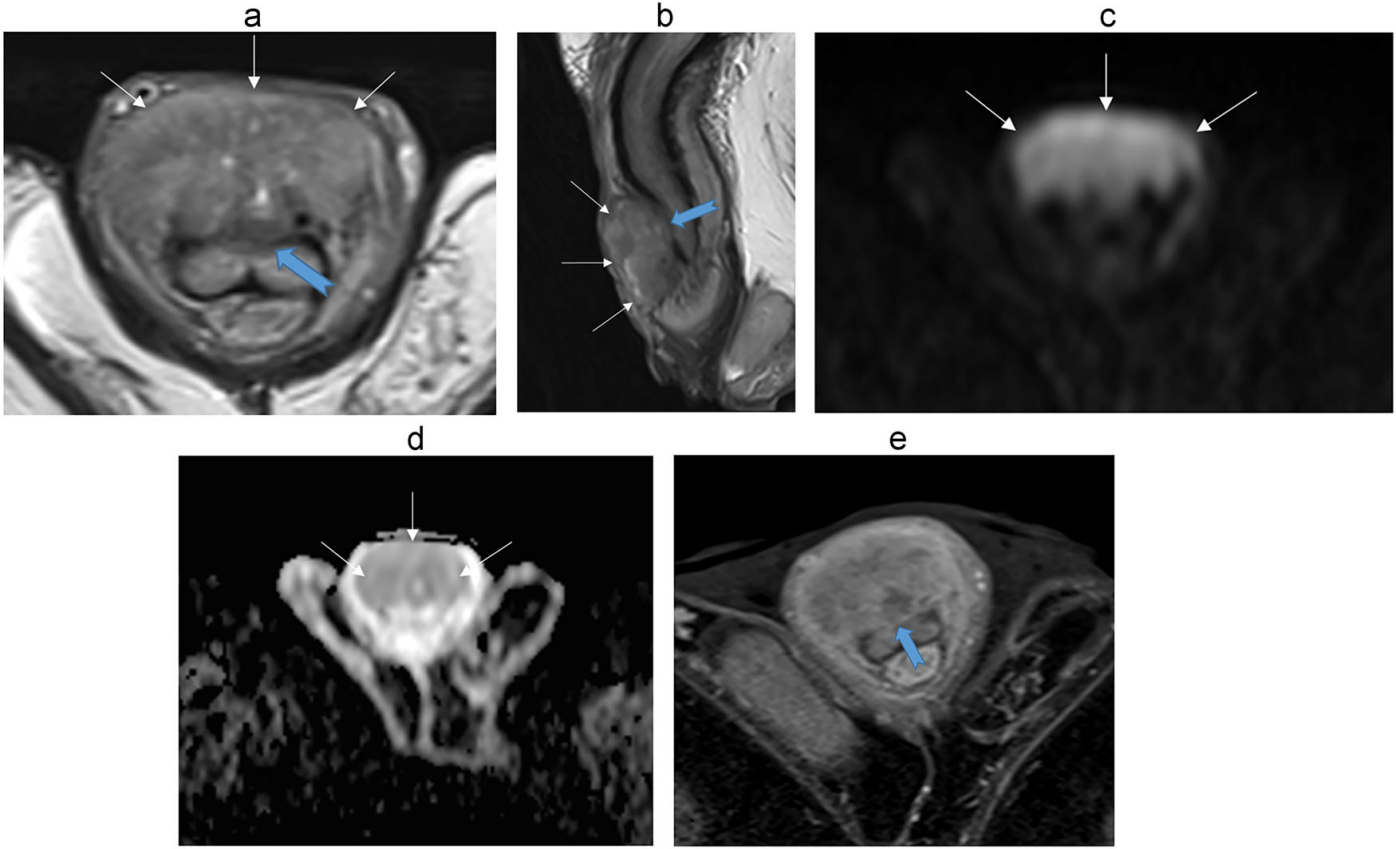
Seventy-five-year-old patient was referred for a large lesion of the glans. **a** Axial, (**b**) sagittal T2-W MR images show a tissue lesion (three white arrows) with intermediate signal. **c** Axial DWI and (**d**) ADC map images show intense diffusion restriction. Disruption of the inner layer of the T2 dark line of albuginea (blue arrow) on axial T2 sequences and on (**e**) enhanced T1 sequence in favor of a cavernosal invasion. Stage pT3N0

**Fig. 6:**
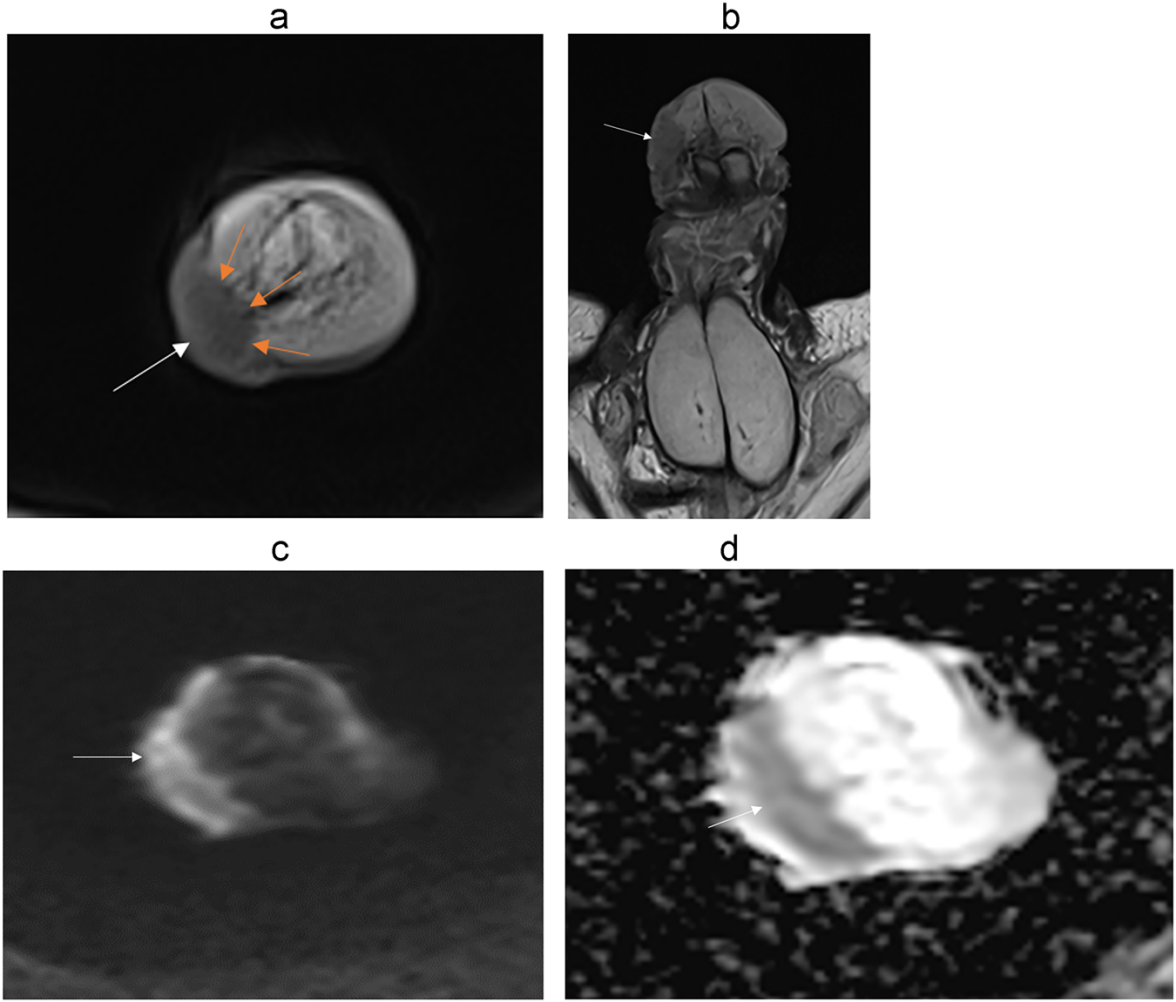
Eighty-three-year-old patient, referred for a cT2 lesion on the right hemi-glans. **a** Axial, (**b**) coronal T2-W MR images show a tissue lesion (with arrow) located on the right lateral ventral surface of the glans, which is hypointense to the corpora, invading the corpus spongiosum (orange arrows). **c** Axial DWI and (**d**) ADC map show intense diffusion restriction. Patient underwent a partial amputation revealing a non-HPV-related epidermoid carcinoma of the papillary type, infiltrating the corpus spongiosum to a maximum height of 0.9 cm. Stage pT2N0

**Fig. 7:**
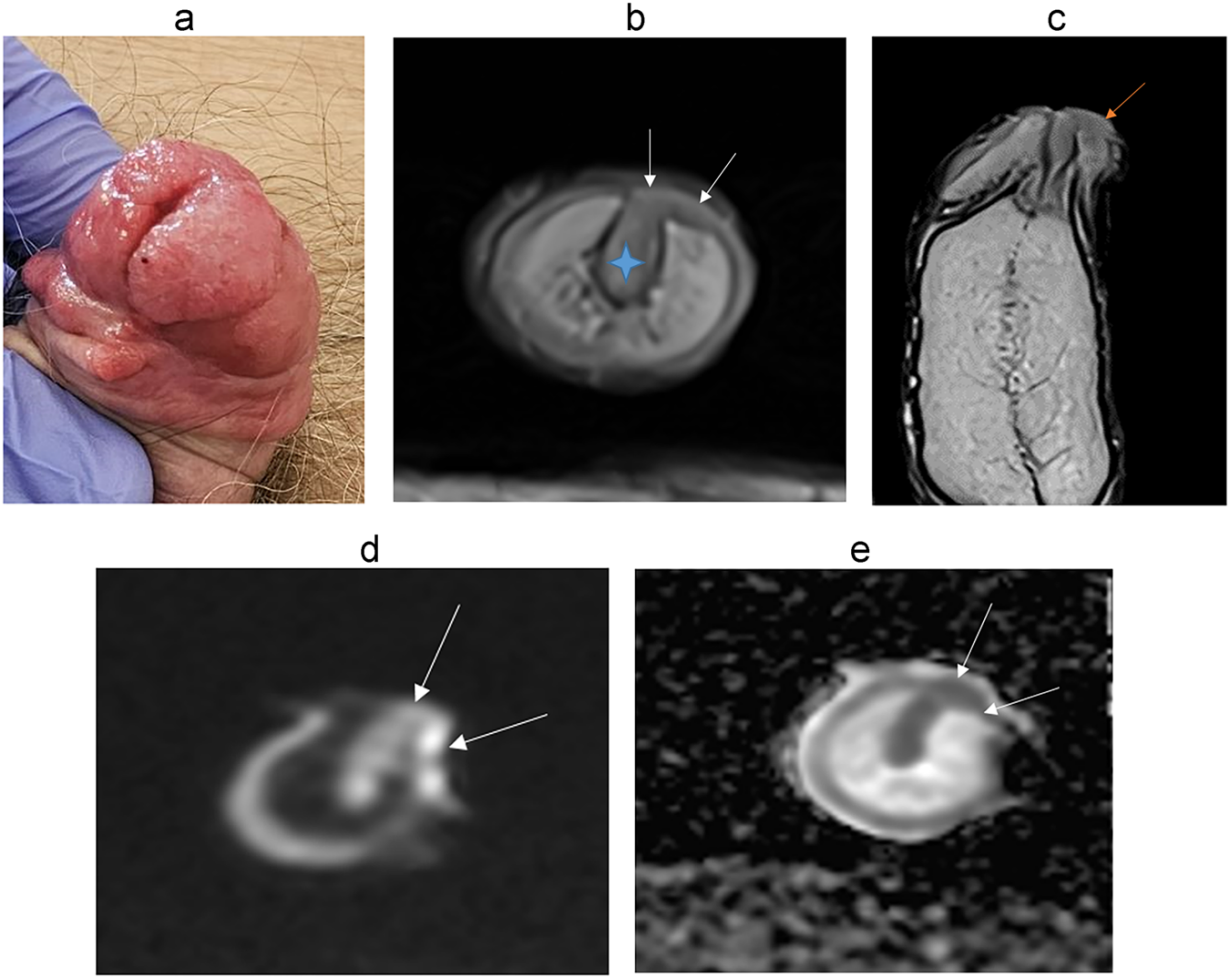
Ninety-one-year-old patient, referred for a cT1 lesion of the glans (**a**). **b** Axial, (**c**) coronal T2-W MR images show a superficial budding lesion of the glans predominant on the left ventral side (white arrows) with distal intra-urethral extension over approximately 8 mm (blue star). There is a millimetric focal invasion of the left corpus spongiosum of the glans on its distal ventral portion (orange arrow). **d** Axial DWI and (**e**) ADC map show intense diffusion restriction. Patient underwent partial distal amputation showing a moderately differentiated HPV-related squamous carcinoma with invasion of the distal penile urethra and infiltrating the corpus spongiosum. Stage pT2N0

**Fig. 8:**
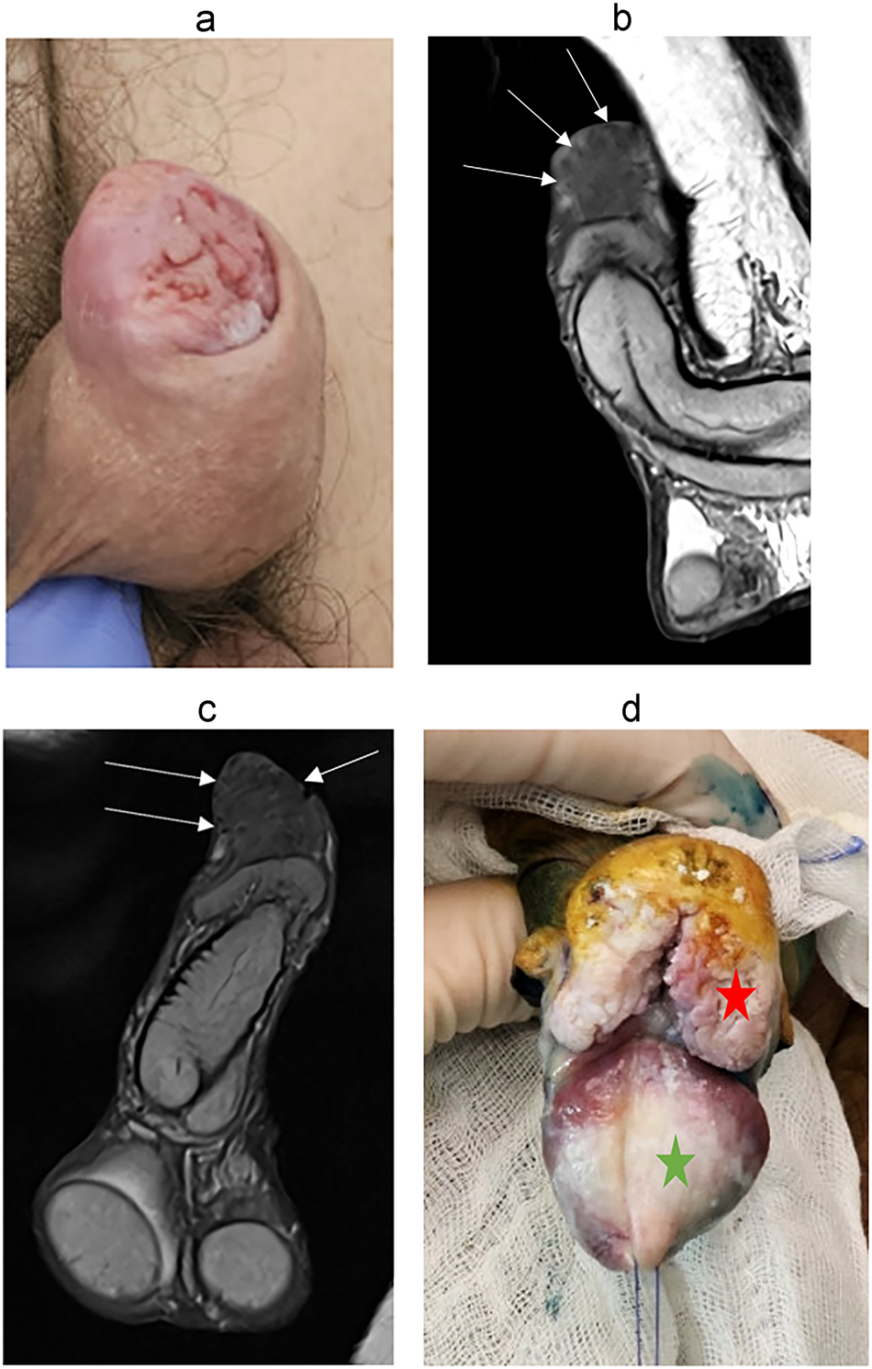
Seventy-two-year-old patient, referred for a malignant-looking tumor on the dorsal part of the foreskin, which comes into contact with the glans and appeared fixed (**a**). **b** Sagittal, (**c**) coronal T2-W MR images show a large budding tumor above the glans, without extension to the corpus spongiosum (arrows). Patient underwent posthectomy showing a well differentiated keratinizing squamous cell carcinoma grade 1 of the foreskin, non HPV associated, limited to the connective planes of the foreskin. Stage pT1N0. Intraoperative image (**d**) showing the tumor excision (red star). The glans appears healthy (green star)

**Fig. 9:**
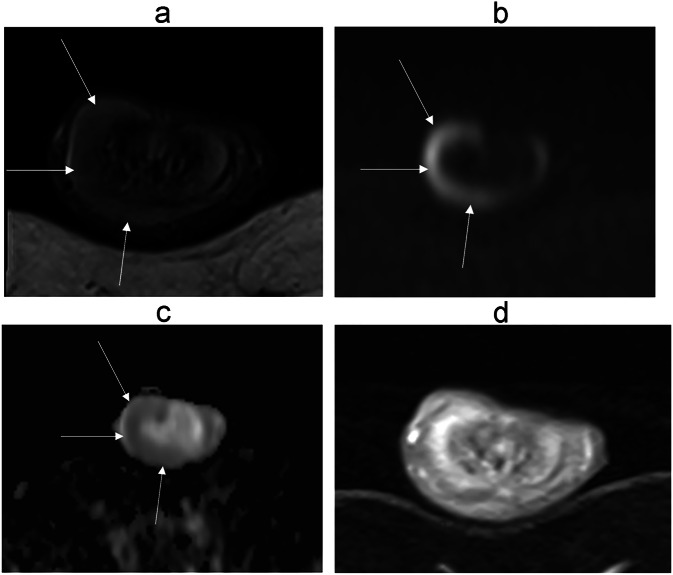
Sixty-seven-year-old patient, consulting in a context of phimosis and penile bleeding. On clinical examination, the lesion is classified as cT2. **a** Axial T2-W MR images, show an extensive tumor with involvement of the corpus spongiosum, predominantly on the right lateral side. **b** Axial DWI and (**c**) ADC map show intense diffusion restriction. The enhanced sequences (**d**) do not provide any further information here. Patient underwent distal partial amputation showing a squamous carcinoma of the glans, grade 2, infiltrating the corpus spongiosum without infiltration of the urethra. Stage pT2N0

**Fig. 10:**
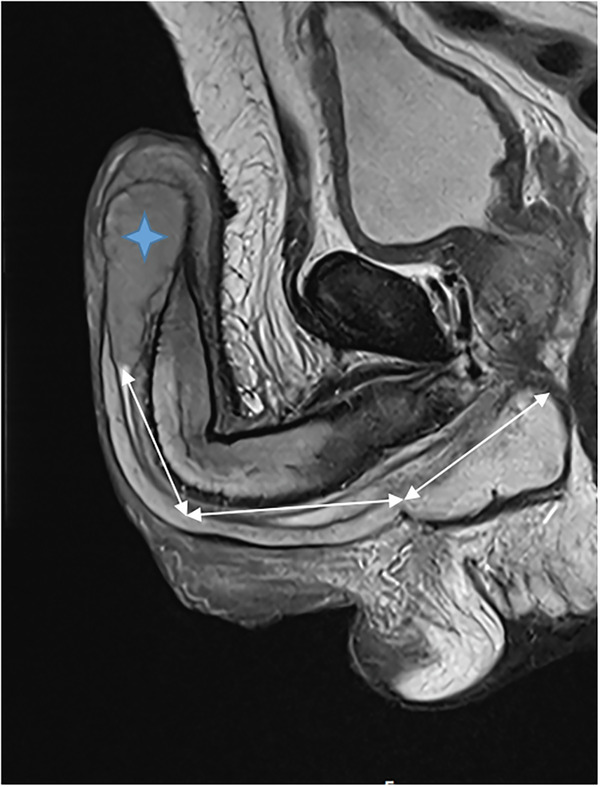
The length of healthy proximal penile tissue, measured from the proximal margin of the tumor to the penoscrotal junction on sagittal T2-weighted images, was here greater than 3 cm. Penile tumor (star)

It is important to assess the depth of infiltration (Figs. 5–10). Since the glans is considered to be the distal part of the corpus spongiosum, a deeply infiltrating tumor of the glans is classified as a T2 disease.

Buck’s fascia and tunica albuginea are identified as a single T2W-hypointense line on MR; however, invasion of Buck’s fascia corresponds to a T1 disease, whereas invasion of tunica albuginea is classified as T3 disease. Involvement of corpus cavernosa (T3 disease) should be diagnosed only when there is unequivocal disruption or interruption of the inner layer of the T2-dark line on MRI (Fig. 5). It should not be overdiagnosed when there is only tumor abutment.

Restricted diffusion within the tumor is typical. One study showed that the mean apparent diffusion coefficient (ADC) of the primary tumor is lower in higher histological grade tumors, with grade 3 tumors exhibiting a mean ADC value of 0.80 × 10^−3^ mm^2^/s and grade 1 tumors having a mean ADC of 0.89 × 10^−3^ mm^2^/s [42].

The tumor appears relatively hypointense on contrast-enhanced MR images, showing a less pronounced enhancement than the corpora cavernosa [18, 28, 31, 37, 43].

In our practice, gadolinium injection is not necessary to define the local extension of penile cancers (Fig. 9). However, it may be useful when findings on unenhanced MRI are equivocal.

#### Lymph node staging

Metastatic disease is usually due to the local lymphatic system, with the Buck fascia acting as a barrier to corporal invasion and hematogenous spread [33]. The lymphatic spread of cancer from the penis depends on tumor location. While tumors of the penile skin and foreskin tend to drain into superficial inguinal lymph nodes, the glans drains into the deep inguinal and external iliac lymph nodes, and the erectile bodies and anterior urethra, into the inguinal, external iliac, or internal iliac lymph nodes [9, 18, 21, 33, 38, 39].

Because the lymphatic vessels communicate with each other, bilateral lymphadenopathy may be seen with a unilateral tumor [18, 21]. The lymphatic vessels of the penis drain into the inguinal nodes before reaching the pelvic nodes [20].

The prevalence of nodal involvement is related to the stage of the primary lesion, the histologic grade, the vertical growth of the tumor, and the presence of vascular or lymphatic invasion [9, 44, 45]. The presence and degree of lymph node involvement are the most important prognostic indicators for patients with penile cancer [3, 34, 42]. Detecting them is crucial for planning treatment strategies.

CT and MRI primarily rely upon lymph node size to assess metastatic involvement. However, this presents a challenge for penile cancer staging.

Conventional imaging techniques (ultrasound, CT, and MRI) cannot distinguish with certainty between tumor invasion and secondary infection, but some authors have found that a necrotic center and lobulated contours are more suggestive of tumor invasion than secondary infection [21, 31, 46]. Lucchesi et al conducted a study which found that MRI was more effective than palpation for nodal staging [47]. ADC values can also be informative and predictive of lymph node metastasis, even in normal-sized nodes, with a cut-off ADC value of < 0.95, as reported in the Barua et al study [42].

MRI helps to determine the need for lymph node dissection by accurately staging the primary cancer. In patients with a clinically negative inguinal exam, the staging of the regional lymph nodes is based on risk estimates for occult nodal metastases.

The latest EAU guidelines [3] recommended performing a physical examination of both groins to assess inguinal nodes and to record the number, laterality, and characteristics of any palpable/suspicious inguinal nodes. If there are no palpable/suspicious nodes (cN0) at physical examination and if the patient is at high risk (T1b and more), dynamic sentinel node biopsy or inguinal lymph node dissection is indicated. If the patient is at lower risk (< T1b), surveillance is recommended rather than nodal assessment.

With a patient with a clinically suspicious inguinal exam, lymph node metastasis should be histopathologically confirmed by image-guided biopsy, before starting therapy.

In cN+ patients, distant metastases should be excluded with ^18^F-fluoro-2-deoxy-D-glucose positron emission tomography (^18^FDG-PET), computed tomography (CT), or CT of the chest and abdomen before initiating treatment. Schlenker et al [48] found that FDG PET/CT showed a sensitivity of 88.2%, a specificity of 98.1%, a positive predictive value of 93.8%, and a negative predictive value of 96.3% in assessing inguinal lymph node involvement in patients with invasive penile carcinoma.

Tips for staging:

- Lymph nodes are often present, but can also reflect secondary infection of an ulcerated lesion.
- Necrotic center and lobulated contours are in favor of tumor invasion.
- Bilateral lymphadenopathy may be present in a unilateral tumor, due to the lymphatic channels' communications between sides.

## Distant metastasis

Hematogenous spread is rare in penile cancer until the later stages of the disease, and distant metastases are seen at presentation in only 2.3–4% of cases [8]. Therefore, routine diagnostic imaging for distant metastases is not recommended. Metastases screening is reserved for selected high-risk patients, including those with bulky regional nodal metastases, due to a higher likelihood of pelvic or retroperitoneal adenopathy, and those presenting with signs and symptoms of metastatic disease. The most common sites of metastases are the lung, liver, retroperitoneum, and bone [18, 38, 39].

Although CT plays only a limited role in primary tumor evaluation, it is the best modality for detecting distant metastatic disease.

## Treatment options

A multidisciplinary team approach with surgeons, oncologists, radiation oncologists, and radiologists is essential for the management of penile cancer.

The treatment plan is primarily guided by the tumor’s size and location, as well as the goal of preserving function. Nevertheless, surgical resection of the primary tumor remains the gold standard, offering the most favorable prognosis.

According to recent literature [3, 49, 50], a surgical margin of 5–10 mm is considered oncologically adequate, with a tumor-free margin of at least 5 mm confirmed by intraoperative fresh frozen section, highlighting the importance of MRI for accurately assessing tumor depth.

Below are the latest updated recommendations according to the EAU guidelines [3].

### Primary tumor

The aim of the treatment of the primary tumor is complete tumor removal, including an adequate tumor-free margin with as much organ preservation as possible.

#### Treatment of superficial non-invasive disease (PeIN)

**Fig. 11:**
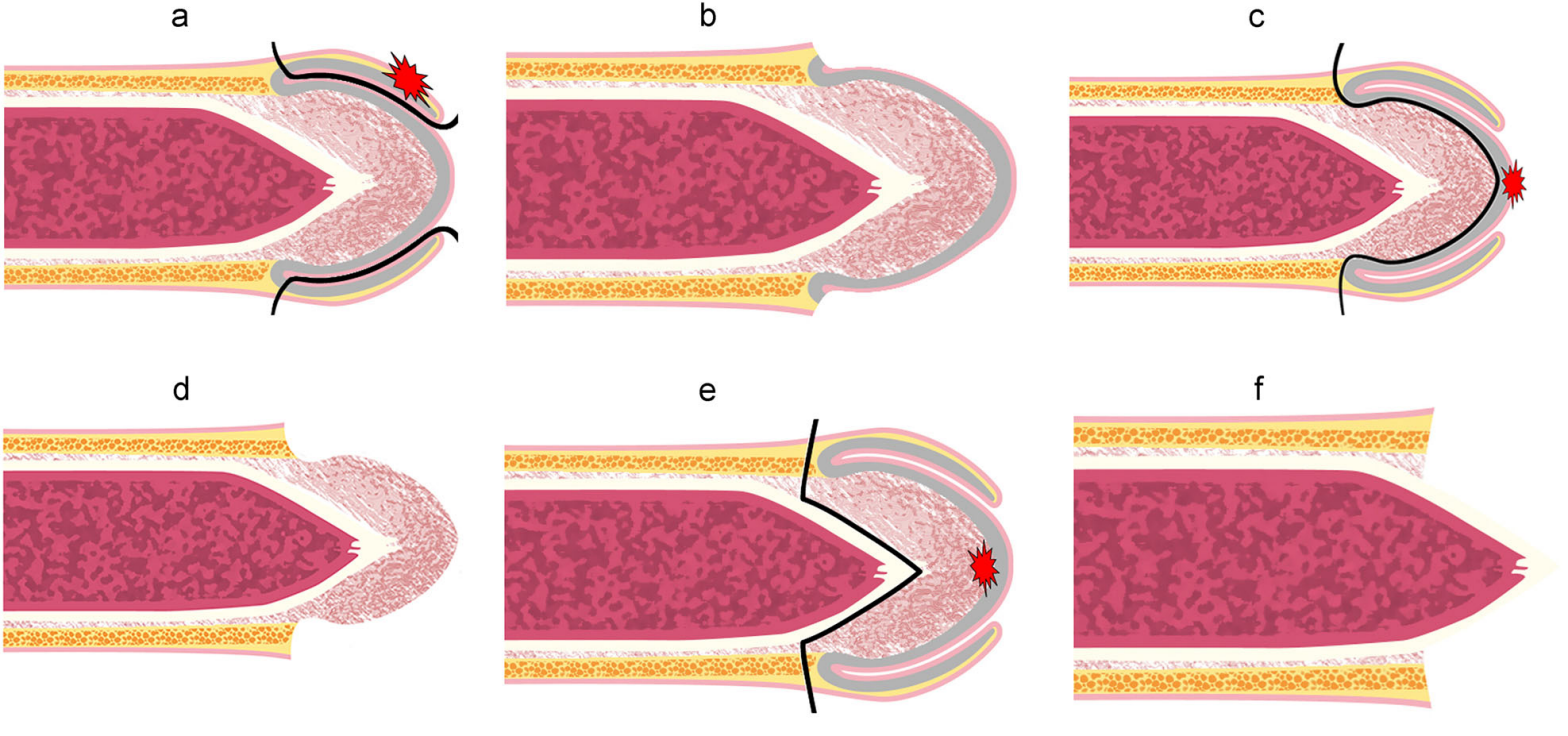
Different surgical techniques. **a**, **b** Circumcision (or posthectomy) consists of removing the foreskin and some nearby skin. **c**, **d** Resurfacing (total or partial): consists of the removal of the glandular epithelium followed by reconstruction with a graft. **e**, **f** Glansectomy (total or partial): consists of removing the glans while maintaining the corpora cavernosa

Circumcision is recommended as the primary surgical option, with close monitoring advised before considering additional treatments (Fig. 11a, b). Other effective treatments include topical chemotherapy with imiquimod or 5-fluorouracil (5-FU) or laser ablation.

Organ-sparing surgery (local excision, glans surfacing (Fig. 11c, d) or glansectomy (Fig. 11e, f), may be used as a primary treatment for PeIN or as a secondary option if topical chemotherapy or laser therapy is unsuccessful.

#### Treatment of invasive disease confined to the glans (T1–T2)

At this stage, organ-sparing treatments with reconstructive techniques should be offered. The various options include circumcision, wide local excision, glans resurfacing, and glansectomy.

Wide local excision is avoided when the tumor is near the urethra, involves the meatus, or extends over more than 50% of the glans penis [50].

Radiotherapy, including EBRT (external beam radiotherapy) or brachytherapy, is also an alternative in selected patients [51, 52].

#### Treatment recommendations for invasive penile cancer (T3–T4)

In patients staged ≥ cT3, (partial or total) amputation surgery is standard. For patients with large invasive tumors that cannot be treated with partial amputation, total penectomy with perineal urethrostomy is recommended.

Partial penectomy involves the removal of the glans with or without part of the underlying corpora cavernosa, leaving behind sufficient length for the patient to stand and void, whereas a total penectomy involves the removal of the glans with the underlying corpora cavernosa without leaving behind enough penile length to enable micturition while standing. Invasion of the corpora cavernosa is an adverse finding after initial organ-sparing treatment that warrants partial or total penectomy [8, 53].

Chemotherapy can be used in patients with non-resectable advanced primary lesions, to induce a treatment response that will allow for subsequent surgery.

### Regional lymph node management

The management of regional lymph nodes is crucial to patient survival.

In patients with cN1 or cN2 disease, radical inguinal lymph node dissection is recommended. A pelvic prophylactic ipsilateral pelvic lymphadenectomy is advised if pathological examination reveals the involvement of three or more inguinal nodes on one side or the presence of extranodal extension.

In patients with cN3 disease, chemotherapy is recommended before lymph node dissection.

Radiotherapy can be added to patients with pN2/N3 disease.

## Surveillance and recurrence

Recurrences generally occur within the first two years of treatment [3]. In a recent study involving 509 patients, it was reported that 52.3% of local recurrences occurred within two years, and 79.5% within three years [54].

In a large retrospective review of 700 patients, Leijte et al [55] reported that the penile-sparing therapy carries a significantly higher risk of local recurrence (28%) than partial or total penectomy (5%). Patients without lymph node involvement had a regional recurrence rate of 2% compared to 19% for patients with lymph node involvement. Thus, they recommended a more intense initial follow-up for patients with lymph node involvement.

A physical examination of the penis and inguinal region is fundamental for the follow-up of all patients. Following penile-preserving treatment, a follow-up visit is recommended every 3 months during the first 2 years, and then every 6 months, including local and regional clinical examination. For N+ patients, imaging of the chest, abdomen, and pelvis using CT or MRI is recommended to detect systemic disease, following the same frequency as the clinical examination [3].

For primary tumor recurrences without infiltration of the corpora cavernosa, salvage penile-sparing options can be considered.

A recurrence in the inguinal region has a poor prognosis (median survival < 6 months), and optimal management remains elusive in this setting [46]. Multimodality treatment combining chemotherapy and surgery or chemoradiation is recommended.

MRI is useful for evaluating local recurrences, while FDG PET/CT is useful for assessing distant metastases, especially when the recurrence is suspected at clinical examinations.

## Other types of cancer

### Penile metastases

Metastases to the penis are rare and originate in about 70% of cases from gastrointestinal (rectal origin) or genitourinary primary cancers, including those of the prostate, bladder, urethra, and testes. The lung is the next most common primary site, accounting for approximately 5% of cases [37, 39, 56–60].

These metastases typically present as either diffuse infiltration of the penis or multiple masses [38, 57]. The spread to the penis can occur through direct infiltration or hematogenous spread.

Metastases frequently involve the corpora cavernosa, but can also invade the spongiosum or the tunica albuginea. In fact, 71% of metastases occur in the penile shaft. This is a distinguishing feature from primary penile carcinoma, which typically occurs in the glans and foreskin [33, 58].

The prognosis of penile metastases is very poor, often indicative of advanced metastatic disease [58].

### Urethral carcinoma (Fig. 12)

Malignancies can also arise directly from the anterior urethra, most commonly of squamous cell origin, but adenocarcinoma and transitional cell carcinoma can also occur [37].

**Fig. 12:**
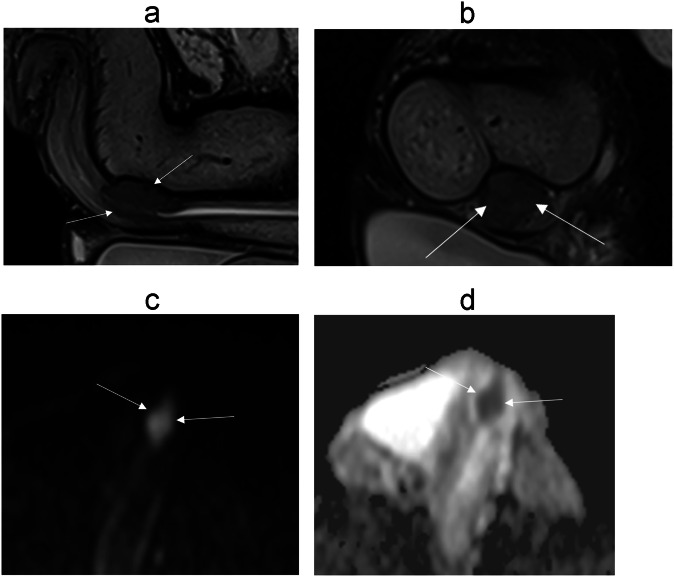
79-year-old patient referred for urethral bleeding. Penile urethral polyps on urethrocystoscopy. Extension assessment requested by MRI. **a** Sagittal, (**b**) axial T2-W MR images show a mass centered on the ureter in hyposignal T2, with invasion of the corpus spongiosum. DWI (**c**) and (**d**) ADC map shows intense diffusion restriction. Patient underwent uretrectomy in favor of an infiltrating urothelial carcinoma infiltrating the periurethral corpus spongiosum without involvement of the corpora cavernosa

MRI is the best imaging modality for local staging of urethral carcinoma [61]. The MRI appearance is similar to that of other penile neoplasms, generally showing low signal on T1, hypointense to intermediate signal on T2, and mild post-contrast enhancement, with restricted diffusion [37, 56]. They most typically arise in the bulbous or membranous urethra, with the epicenter of the lesion located along the urethra or the corpus spongiosum [37, 38, 58], distinguishing them from other penile tumors. The mass lesion may obstruct the proximal urethra and result in urinary retention [33]. The penile urethra drains into the superficial and deep inguinal lymph nodes, while the bulbous, membranous, and prostatic urethra drain into deep pelvic lymph nodes [58, 61]. Local nodal metastases are best staged using either CT or MRI. Clinically palpable lymph nodes are usually metastatic in urethral carcinoma [61]. These tumors are typically very aggressive, with distant metastases most commonly affecting the liver or the lung [58].

### Penile lymphoma

Penile lymphoma is a very rare neoplasm, typically appearing T1 and T2 hypointense and showing low-level enhancement following contrast [62]. Involvement of the penis may be due to direct invasion from an adjacent nodal lesion, or to hematogenous or lymphatic spread [62].

### Sarcoma

Sarcomas can also rarely arise in the penis. Subtypes include epithelioid sarcoma, leiomyosarcoma, rhabdomyosarcoma, and Kaposi sarcoma. Collectively, these tumors account for approximately 5% of all penile malignancies [14, 41].

Sarcomas are often large masses, locally invasive, with heterogeneous signal on T1-weighted images, hyperintensity on T2-weighted images, diffusion restriction, and heterogeneous or peripheral enhancement after contrast [56, 63].

Epithelioid sarcoma and leiomyosarcoma have both been reported to clinically mimic Peyronie’s disease, with a palpable nodule and penile curvature [63].

Penile rhabdomyosarcoma can occur in pediatric patients [41].

A few cases have been reported of Kaposi’s sarcoma of the penis in Human immunodeficiency virus (HIV) patients, but also in HIV-negative patients [64, 65].

## Conclusion

Primary carcinomas of the penis are mainly found on the glans and foreskin, with HPV infection one of the major risk factors. Palpation alone for assessing a primary penile tumor and inguinal lymph nodes can lead to inaccurate staging.

MRI, when used with an appropriate technique, provides the most accurate preoperative assessment of the depth of the local tumor extension within the corpus spongiosum or the corpora cavernosa. The most informative sequences are the T2-weighted sequences. Assessment of lymph node involvement, a major prognostic factor in penile cancer, remains a limitation of sectional imaging.

Therefore, an accurate staging of penile cancer requires a combination of physical examination, imaging studies including FDG PET/CT, and lymph node biopsies when needed.

The precise assessments of the primary tumor’s extent and the detection of locoregional and distant metastases are crucial to treatment planning.

## Abbreviations

ADC: Apparent diffusion coefficient
EAU: European Association of Urology
HIV: Human immunodeficiency virus
HPV: Human papillomavirus
MRI: Magnetic resonance imaging
PeiN: Penile intraepithelial neoplasia
SCC: Squamous cell carcinoma
TNM: Tumor-node-metastasis
UICC-AJCC: Union for International Cancer Control (UICC)/American Joint Committee on Cancer (AJCC)
